# *SMAD3* Is Associated with the Total Burden of Radiographic Osteoarthritis: The Chingford Study

**DOI:** 10.1371/journal.pone.0097786

**Published:** 2014-05-22

**Authors:** Erfan Aref-Eshghi, Yuhua Zhang, Deborah Hart, Ana M. Valdes, Andrew Furey, Glynn Martin, Guang Sun, Proton Rahman, Nigel Arden, Tim D. Spector, Guangju Zhai

**Affiliations:** 1 Faculty of Medicine, Memorial University of Newfoundland, St. John's, Newfoundland, Canada; 2 Department of Twin Research & Genetic Epidemiology, King's College London, London, United Kingdom; 3 Musculoskeletal Epidemiology and Biobank, University of Oxford, Oxford, United Kingdom; National Cancer Center, Japan

## Abstract

**Background:**

A newly-described syndrome called Aneurysm-Osteoarthritis Syndrome (AOS) was recently reported. AOS presents with early onset osteoarthritis (OA) in multiple joints, together with aneurysms in major arteries, and is caused by rare mutations in *SMAD3*. Because of the similarity of AOS to idiopathic generalized OA (GOA), we hypothesized that *SMAD3* is also associated with GOA and tested the hypothesis in a population-based cohort.

**Methods:**

Study participants were derived from the Chingford study. Kellgren-Lawrence (KL) grades and the individual features of osteophytes and joint space narrowing (JSN) were scored from radiographs of hands, knees, hips, and lumbar spines. The total KL score, osteophyte score, and JSN score were calculated and used as indicators of the total burden of radiographic OA. Forty-one common SNPs within *SMAD3* were genotyped using the Illumina HumanHap610Q array. Linear regression modelling was used to test the association between the total KL score, osteophyte score, and JSN score and each of the 41 SNPs, with adjustment for patient age and BMI. Permutation testing was used to control the false positive rate.

**Results:**

A total of 609 individuals were included in the analysis. All were Caucasian females with a mean age of 60.9±5.8. We found that rs3825977, with a minor allele (T) frequency of 20%, in the last intron of *SMAD3*, was significantly associated with total KL score (β = 0.14, P_permutation_ = 0.002). This association was stronger for the total JSN score (β = 0.19, P_permutation_ = 0.002) than for total osteophyte score (β = 0.11, P_permutation_ = 0.02). The T allele is associated with a 1.47-fold increased odds for people with 5 or more joints to be affected by radiographic OA (P_permutation_ = 0.046).

**Conclusion:**

We found that *SMAD3* is significantly associated with the total burden of radiographic OA. Further studies are required to reveal the mechanism of the association.

## Introduction

Osteoarthritis (OA) is the most common form of arthritis in the elderly, characterized pathologically by focal areas of damage to the articular cartilage centered on load-bearing areas. It is associated with new bone formation at the joint margins (osteophytosis), changes in the subchondral bone, variable degrees of mild synovitis, and thickening of the joint capsule [1] which lead to the presentation of pain, stiffness and disability. Its prevalence—already high—is increasing due to population aging and the increase in obesity. Eighty percent of individuals over 75 years of age have radiographic OA changes in at least one of their joints [2]. According to a report from the Arthritis Community Research & Evaluation Unit in April 2010, the prevalence of self-reported and physician-diagnosed OA in individuals over age 45 ranged from 2.3%–11% in the third world to 8%–16% in the USA [3]. In the same year it affected 27 million people in the USA, imposing a burden of over 11 million dollars on outpatient visits and over 13 billion dollars on OA-related job absence [4]. Half of all adults will develop symptomatic OA of the knee at some points in their lives [5].

OA is a multifactorial disease whose etiology is incompletely understood. It is believed that a number of different environmental and genetic factors interact in its initiation and progression [6]. Evidence suggests that genetic factors play a major role in OA, although they may be site- and sex-specific. From twin studies, this genetic influence has been estimated to be between 40% and 65% on hand and knee OA [7]. First-degree relatives of individuals with spine, hand, hip, or polyarticular OA have a two- to three-fold increased risk of the disease [8], [9]. The nature of the genetic influence in OA is still unclear but it is likely to involve a combination of effects on structure (i.e. collagen), alterations in cartilage, bone metabolism and inflammation [10]. Although the genetic influence on OA was recognized more than 130 years ago [11], genetic variants identified so far account only for a small fraction of its heritability [12]. This may reflect several factors including the heterogeneous nature of the disease, the tendency to use less severe phenotypes in genetic searches and the reliance on underpowered studies [13]. Generalized OA—a subtype of primary OA—is characterized by the involvement of multiple joints, and is believed to have a stronger genetic component than individual joint OA [14]. However, genetic data on generalized OA are limited.

Recently, a new syndrome called Aneurysm-Osteoarthritis Syndrome (AOS) was reported [15]. Patients with AOS present with early-onset OA affecting multiple joints including feet/ankle, hand/wrist, knee, hip, facet joints, uncovertebral joints and also exhibit degeneration of the intervertebral discs [15], [16]. Eight rare mutations in the *SMAD3* gene (Similar to Mothers Against Decapentaplegic type 3) were identified as responsible for AOS in eight unrelated families.^15, 16^ Subsequent studies reported additional *SMAD3* mutations [17], [18] and also a CNV (copy number variant) [19] linked to AOS. The *SMAD3* gene encodes a protein that belongs to the SMAD protein family, that are downstream mediators of the transforming growth factor beta (*TGF-β*) signaling pathway [20], which inhibits terminal hypertrophic differentiation of chondrocytes and is essential for maintaining the integrity of articular cartilage [20], [21]. This regulatory pathway also stimulates osteogenesis and bone formation [22]. *SMAD3* knock-out mice develop degenerative joint disease similar to human OA [23]. Although a few studies on *SMAD3* and single-joint OA have been reported, no data are available regarding the role *SMAD3* plays in generalized OA. Because of the similarity with AOS, in which multiple joints are also affected, we hypothesized that the *SMAD3* gene plays a role in idiopathic generalized OA. We tested our hypothesis in a large population-based cohort of individuals who had radiographic assessment of multiple joints.

## Methods and Subjects

### Subjects

The study subjects were women aged 43-67 years at baseline (1988–1989) who were participating in the Chingford Study, a prospective population-based study of OA and osteoporosis. The Chingford Study cohort comprises 1003 women derived from the register of a large general practice in North London, who are similar to the UK population for most demographic variables [24].

Height, weight and details of concomitant diseases, operations and medications were recorded for all subjects. DNA was extracted from blood by standard phenol or salting-out methods. At both baseline and 10 years later, all subjects completed a standardized medical history questionnaire.

### Ethics

The Guys & St Thomas' Trust and the Waltham Forest Trust ethics committees approved the study protocol. Written consent was obtained from all participants.

### Radiography

Plain films of all joints were obtained from a standard postero-anterior view at baseline and again 9–11 years later. The distal interphalangeal (DIP), proximal interphalangeal (PIP) and first carpometacarpal (CMC) joints of the thumbs, the knee- and hip-joints, as well as four lumbar spinal joints (L1–L5) were assessed for radiographic OA according to the Kellgren & Lawrence (KL) score using a 0–4 scale [25]. Joint Space Narrowing (JSN) and osteophyte characteristics were each scored on a 0–3 scale using a standard atlas [26]. All radiographs were independently assessed by two trained observers (DJ Hunter and DJ Hart). In cases of disagreement, a third adjudicator was used. The intra- and inter-observer reproducibility of the scoring measured on a subgroup of 50 hands had a Kappa statistic of approximately 0.68 for all sites and features.

For the current study, the most recent radiographic and demographic data were used, including cross sectional radiographic data for hip, spine, knee and hand from years 8, 9, 10, and 11 and the age and body mass index (BMI) from the 8^th^ year of the study. All patients were visited at year 8, when the demographic information was collected. Due to the schedule of the radiology department, different joints were assessed in different years (between year 8 and year 11). Total KL score, osteophyte and JSN scores were used as indicators of the total burden of radiographic OA, which was calculated by summing up the individual scores of each joint. Total radiographic scores have been used by researchers in clinical, biomedical, and genetic studies of OA [14], [27]–[29] as an indicator for total burden of OA. In addition, individuals were evaluated for the criteria required for a diagnosis of generalized OA. To this end, joints were defined as being affected by OA if the KL score was ≥2. OA of either the DIP or PIP joint groups was defined as the presence of OA in at least two of the relevant joints. A diagnosis of GOA was based on the definition used by Cooper et al [30]. Fourteen joints or joint groups were considered: the four lumbar joints together with the left and right knee, hip, DIP group, PIP group and thumb CMCs. GOA was defined as the presence of OA in at least five of these 14 joints. Those with fewer than five joints affected were designated as controls.

### Genotyping

The samples were genotyped using the Illumina HumanHap610Q array. The normalized intensity data was used by the Illluminus calling algorithm [31] to assign genotypes. No calls were assigned if an individual's most likely genotype was called with a posterior probability threshold of less than 0.95. Sample exclusion criteria were: (i) sample call rate <98%, (ii) heterozygosity across all SNPs ≥2 s.d. from the sample mean; (iii) evidence of non-European ancestry as assessed by PCA comparison with HapMap3 populations; (iv) observed pair-wise IBD probabilities suggestive of sample identity errors; (v). SNP exclusion criteria included (i) Hardy-Weinberg p-value<10^−6^, assessed in a set of unrelated samples; (ii) MAF<1%, assessed in a set of unrelated samples; (iii) SNP call rate <97% (SNPs with MAF≥5%) or <99% (for 1%≤MAF<5%). For the current study, we retrieved genotype data for all 41 SNPs within the *SMAD3* gene which were available on the array.

### Statistics

Since the distribution of the total KL, JSN, and osteophyte scores was skewed, a logarithmic transformation was performed to approximate a normal distribution. Subsequent analyses were performed on the log-transformed values. A linear regression model, testing for an additive genetic model, was used to test the association between each of the 41 candidate SNPs and the total KL, JSN, and osteophyte scores individually. A logistic regression model was used to test the association between each of the 41 SNPs and GOA. Potential confounders such as age and BMI were considered in both models. All SNP associations with p<0.05 in the initial analyses were subject to permutation testing in order to control the false positive rate. The permutation method is well established as a robust approach for obtaining empirical significance levels while minimizing Type I errors [32], [33], and has been used to correct for multiple testing in genetic association studies [34]. Because of the infinite permutation with our sample size, we used a Monte Carlo permutation procedure and the phenotype labels were reshuffled 10,000 times. The permutation-based p-value was calculated as the proportion of the statistic on all the reshuffled data sets greater than the observed statistic [34]. The significance level was defined as a permutation-based p-value of less than 0.05. All analyses were conducted using STATA/SE 11.2 (Stata Corp, College Station, Texas, USA).

## Results

All study subjects were Caucasian females. Radiographic data for spine, hips, knees and hand joints were available for 796, 794, 614, and 687 individuals, respectively. The age and BMI data were available for 843 participants with a mean age of 61.2±5.8 and a mean BMI of 26.7±4.7. Total KL, osteophyte and JSN scores were available for 609, 603, and 607 individuals respectively. As expected, patients with GOA—defined as having 5 or more joints affected—were, on average, older than those with fewer than 5 affected joints, and also had a higher BMI (Table 1). The frequency of subjects with different number of affected joints is presented in Table 2.

**Table 1:** Descriptive statistics of the study population.

	GOA (n = 247)	Controls (n = 360)	P-Value
**Age**	64.21±0.34	58.71±0.3	P<0.0001
**BMI**	27.50±0.26	26.02±0.2	P<0.0001

Figures are mean ± SD, and Student's T-test was used for the comparison.

**Table 2:** Frequency of patients with different number of joints affected.

Number of Joints affected	Frequency (%)
**0**	43 (7.06%)
**1**	62 (10.18%)
**2**	83 (13.63%)
**3**	93 (15.27%)
**4**	80 (13.14%)
**5**	51 (8.37%)
**6**	64 (10.51%)
**7**	38 (6.24%)
**8**	36 (5.91%)
**9**	22 (3.61%)
**10**	15 (2.46%)
**11**	12 (1.97%)
**12**	9 (1.48%)
**13**	1 (0.16%)
**Total**	609 (100%)

**Figure 1:**
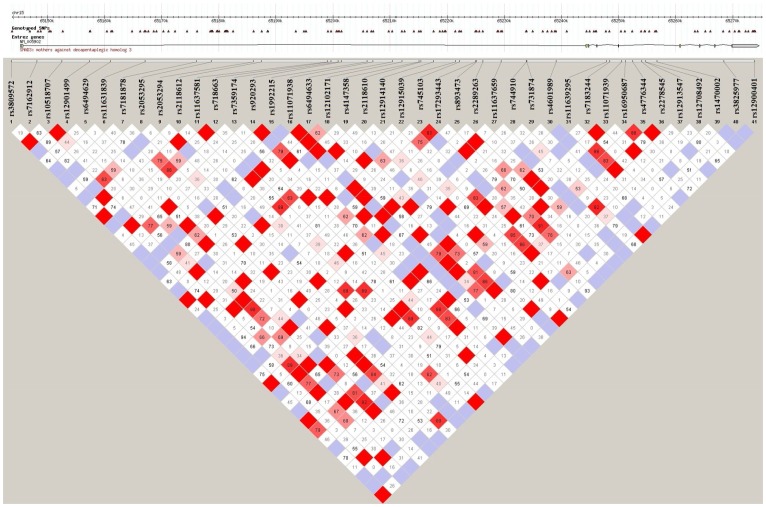
Distribution and LD pattern of 41 genotyped SNPs in SMAD3 gene.

Forty-one common SNPs within the *SMAD3* gene were genotyped and passed quality control. They were scattered randomly throughout the *SMAD3* gene, but none were located in exons (Figure 1). The average pairwise R^2^ between SNPs was 0.07.

**Figure 2:**
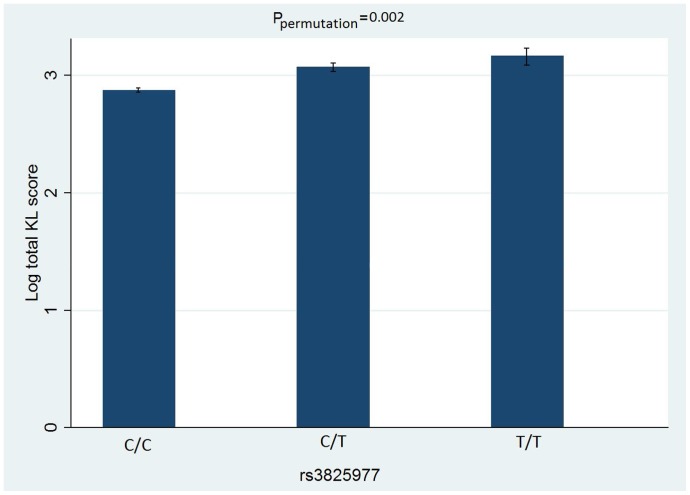
Total KL and each genotype of rs3825977. Error bars indicate Standard Error of the mean.

**Figure 3:**
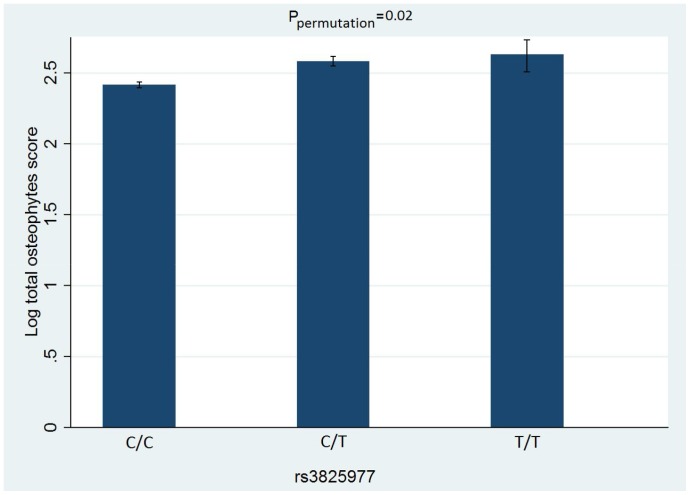
Total osteophyte and each genotype of rs3825977. Error bars indicate Standard Error of the mean.

**Figure 4:**
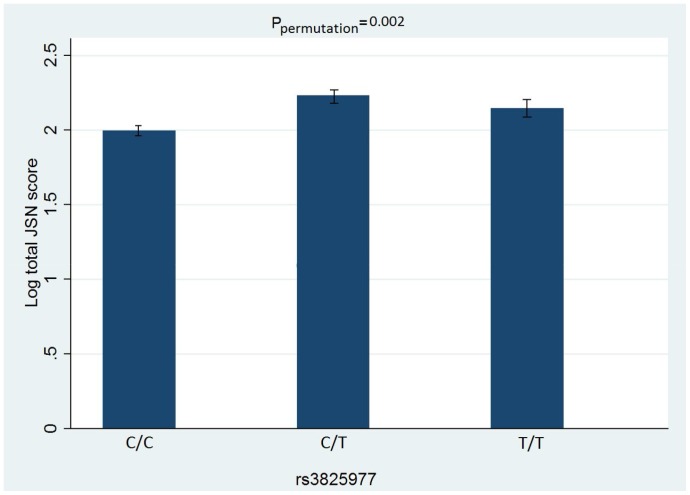
Total JSN and each genotype of rs3825977. Error bars indicate Standard Error of the mean.

We found that SNP rs3825977 was significantly associated with all phenotypes analyzed, *viz*. total KL, osteophyte and JSN scores. The differences in these traits among individuals with different genotypes are presented in Figures 2, 3, 4. After adjustment for age and BMI, the minor (T) allele of rs3825977—with 20% allele frequency—was associated with a 0.14% increase in log total KL score (95% CI 0.04–0.20, P_permutation_ = 0.002). The association is stronger for log total JSN score with β = 0.19 (95%CI 0.07–0.31, P_permutation_ = 0.002) than for log total osteophyte score with β = 0.11 (95%CI 0.01–0.20, P_permutation_ = 0.02). Two other SNPs—rs6494629 and rs2118612—were significant for only total osteophyte score in the univariate analysis but not in a multivariate analysis. All the results of univariate and multivariate linear regression analyses for total KL, osteophyte and JSN scores for all 41 SNPs are presented in Tables S1–S3 in File S1, respectively.

Furthermore, we categorized the study participants into two groups: one with ≥5 joints affected (GOA) and one with <5 joints affected and examined the association of each group with each of the 41 SNPs. We found that the T allele of rs3825977 was significantly associated with a 1.47-fold increased risk of GOA (95% CI 1.02–2.1, P_permutation_ = 0.046) after adjustment for age and BMI (Table 3). All results of the associations with each of the 41 SNPs are presented in Table S4 in File S1.

**Table 3:** Association between GOA and rs3825977*.

	Multivariable Analysis			MAF	
Variables	OR (95% CI)	P-value	P_permutation_	Cases	Controls
**rs3825977 (T vs. C allele)**	1.47 (1.02–2.1)	0.037	0.046	0.23	0.17
**Age (per year)**	1.2 (1.16–1.25)	<0.0001	-	-	-
**BMI (per kg/m^2^)**	1.09 (1.04–1.15)	<0.0001	-	-	-

*Logistic regression was used. MAF: Minor allele frequency, OR: Odds Ratio, CI: Confidence interval.

## Discussion

In the present study we demonstrate a significant association of SNP rs3825977—located in the last intron of *SMAD3*—with the total burden of radiographic OA. This SNP is more strongly associated with total JSN score than with total KL score or osteophyte score, suggesting that the potential mechanism for the association is more likely through cartilage loss rather than osteophyte formation. The same SNP has previously been reported as associated with increased breast cancer risk for *BRCA2* mutation carriers [35]. Although the possible effect of the SNP on *SMAD3* function is still unclear, it is believed that the effects on both breast cancer and generalized OA susceptibility are mediated through the *TGF-β* signaling pathway.

Data on the associations between the *SMAD3* gene and GOA are limited and, to our knowledge, no genetic or genome-wide association study has been performed on GOA. A study by Yao JY, *et al.* [36] was the first to report a connection between *SMAD3* and OA. This paper described a missense mutation located in the linker region of the SMAD3 protein which resulted in an increased expression of matrix metalloproteinase (MMP) 2 and 9 in the serum of one OA mutation carrier compared to MMP expression in other OA patients and in controls. Another study by A. Valdes and colleagues [37] reported the association of a variant in the *SMAD3* gene with hip and knee OA. In that study the frequency of the major (G) allele of rs12901499—located in the first intron of *SMAD3*—was increased in patients undergoing hip or knee replacement as compared to controls. A recent study by Jiang Liying *et al*. [38] found this SNP was also associated with hand and knee OA in a northeast Chinese population. However, we did not observe a significant association with rs12901499, which is not in LD with rs3825977 (R^2^ = 0.01). This may have resulted from the different methods used for the definition and classification of OA in our study and the previous studies which used either end-stage OA (requiring total joint replacement) or symptomatic OA, neither of which is necessarily concordant with radiographic OA [39]. Alternatively, one or both of these SNPs may be non-functional but rather in LD with causal variants in the gene that were not typed in these studies.

Cartilage homeostasis depends on a balance between the catabolic and anabolic activities of chondrocytes being controlled by numerous cytokines and growth factors. *TGF-β* is an important molecule that plays a critical role in the development, growth, maintenance and repair of articular cartilage by modifying the metabolism of the chondrocyte. Deregulation of *TGF-β* signaling and responses have been shown to be involved in OA [20]. The SMAD family proteins, including SMAD3, are important intracellular signals in the *TGF-β* pathway [21]. Another possible mechanism by which SMAD3 acts to maintain cartilage homeostasis is by inducing the expression of type II collagen and repressing *MMP-13*. A recent study by Chen and colleagues [40] showed that *SMAD3* ^(fl/fl)^ mice were severely deficient in both type II collagen and aggrecan due to the proteolytic activity of *MMP-13*, which is normally down-regulated by *TGF-β* signals mediated through *SMAD3*.

There are some limitations in the study. All the participants were female, which limits the generalizability. Given its unknown function, it is not clear whether the associated SNP is causal.

## Conclusions

We demonstrated that the *SMAD3* gene is associated with the total burden of radiographic OA. As a marker, it has a potential in identifying those with increased risk of OA, thus permitting earlier joint-preserving intervention. It also has potential as a molecular target for developing new OA drugs.

## Supporting Information

File S1 **Contains the files: Table S1** Univariate and multivariate linear regression for total KL score and each SNP. **Table S2** Univariate and multivariate linear regression for total osteophytes score and each SNP. **Table S3** Univariate and multivariate linear regression for total JSN score and each SNP. **Table S4** Univariate and multivariate logistic regression for GOA and each SNP.
(DOC)
