# Supplementary material for: SMAD3 Is Associated with the Total Burden of Radiographic Osteoarthritis: The Chingford Study
Source: PLoS One. 2014 May 22;9(5):e97786. doi: 10.1371/journal.pone.0097786 (PMC4031234; doi:10.1371/journal.pone.0097786)
Supplement: File S1 — Contains the files: Table S1 Univariate and multivariate linear regression for total KL score and each SNP. Table S2 Univariate and multivariate linear regression for total osteophytes score and each SNP. Table S3 Univariate and multivariate linear regression for total JSN score and each SNP. Table S4 Univariate and multivariate logistic regression for GOA and each SNP. (DOC) [file pone.0097786.s001.doc]

Table S1- Univariate and multivariate linear regression for total KL score and each SNP

|  | **Univariate Analysis** | | **Multivariate Analysis** | |
| --- | --- | --- | --- | --- |
| **SNP** | **Coefficient (95% CI)** | **P-value** | **Coefficient (95% CI)** | **P-value** |
| **rs3825977** | 0.178 (0.068 - 0.288) | 0.002 | 0.143 (0.048 - 0.238) | 0.003 |
| **rs6494629** | -0.085 (-0.171 - 0.001) | 0.053 | -0.051 (-0.125 - 0.023) | 0.179 |
| **rs745103** | -0.052 (-0.137 - 0.034) | 0.237 | -0.05 (-0.123 - 0.024) | 0.183 |
| **rs718663** | -0.069 (-0.274 - 0.137) | 0.513 | -0.102 (-0.278 - 0.075) | 0.259 |
| **rs11637659** | -0.054 (-0.166 - 0.059) | 0.351 | -0.058 (-0.155 - 0.039) | 0.241 |
| **rs2118612** | -0.091 (-0.206 - 0.024) | 0.119 | -0.058 (-0.157 - 0.041) | 0.253 |
| **rs16950687** | 0.044 (-0.051 - 0.14) | 0.358 | 0.039 (-0.043 - 0.121) | 0.348 |
| **rs11631839** | 0.05 (-0.037 - 0.136) | 0.257 | 0.034 (-0.04 - 0.109) | 0.365 |
| **rs12900401** | -0.115 (-0.318 - 0.088) | 0.266 | -0.079 (-0.254 - 0.096) | 0.374 |
| **rs893473** | -0.06 (-0.176 - 0.056) | 0.307 | -0.043 (-0.143 - 0.057) | 0.400 |
| **rs744910** | -0.027 (-0.115 - 0.061) | 0.547 | -0.028 (-0.104 - 0.048) | 0.477 |
| **rs1470002** | 0.069 (-0.023 - 0.16) | 0.143 | 0.026 (-0.053 - 0.106) | 0.513 |
| **rs2278545** | -0.042 (-0.188 - 0.103) | 0.567 | -0.04 (-0.165 - 0.086) | 0.535 |
| **rs11071939** | -0.082 (-0.245 - 0.081) | 0.323 | -0.047 (-0.187 - 0.093) | 0.513 |
| **rs2118610** | 0.016 (-0.07 - 0.103) | 0.711 | 0.023 (-0.053 - 0.098) | 0.558 |
| **rs11637581** | 0.012 (-0.08 - 0.104) | 0.799 | 0.025 (-0.055 - 0.104) | 0.537 |
| **rs2053295** | 0.068 (-0.063 - 0.2) | 0.307 | 0.036 (-0.078 - 0.149) | 0.537 |
| **rs12914140** | -0.056 (-0.231 - 0.118) | 0.525 | -0.051 (-0.201 - 0.099) | 0.506 |
| **rs920293** | 0.042 (-0.101 - 0.186) | 0.562 | 0.037 (-0.088 - 0.161) | 0.563 |
| **rs4601989** | 0.055 (-0.052 - 0.163) | 0.314 | 0.026 (-0.067 - 0.119) | 0.585 |
| **rs6494633** | 0.015 (-0.072 - 0.102) | 0.742 | 0.022 (-0.054 - 0.098) | 0.566 |
| **rs2053294** | 0.059 (-0.073 - 0.191) | 0.382 | 0.031 (-0.083 - 0.145) | 0.595 |
| **rs12915039** | 0.031 (-0.075 - 0.138) | 0.563 | 0.024 (-0.068 - 0.116) | 0.608 |
| **rs4147358** | -0.037 (-0.145 - 0.071) | 0.503 | -0.021 (-0.115 - 0.072) | 0.654 |
| **rs7183244** | -0.033 (-0.12 - 0.054) | 0.455 | -0.018 (-0.093 - 0.057) | 0.645 |
| **rs7359174** | -0.078 (-0.217 - 0.06) | 0.267 | -0.025 (-0.144 - 0.094) | 0.679 |
| **rs2289263** | 0.04 (-0.044 - 0.123) | 0.352 | 0.013 (-0.059 - 0.086) | 0.723 |
| **rs17293443** | -0.007 (-0.11 - 0.096) | 0.897 | -0.016 (-0.105 - 0.072) | 0.722 |
| **rs3809572** | -0.008 (-0.141 - 0.124) | 0.900 | -0.02 (-0.134 - 0.095) | 0.734 |
| **rs12913547** | 0.08 (-0.029 - 0.189) | 0.151 | 0.017 (-0.078 - 0.111) | 0.732 |
| **rs7181878** | -0.038 (-0.124 - 0.049) | 0.390 | -0.011 (-0.086 - 0.064) | 0.772 |
| **rs11071938** | 0.007 (-0.087 - 0.1) | 0.884 | -0.01 (-0.091 - 0.071) | 0.803 |
| **rs11639295** | 0.016 (-0.08 - 0.112) | 0.748 | -0.011 (-0.094 - 0.072) | 0.795 |
| **rs1992215** | 0.011 (-0.083 - 0.104) | 0.825 | -0.009 (-0.09 - 0.072) | 0.82 |
| **rs12901499** | 0.001 (-0.087 - 0.088) | 0.989 | -0.006 (-0.081 - 0.07) | 0.886 |
| **rs731874** | -0.02 (-0.11 - 0.07) | 0.658 | 0.005 (-0.074 - 0.083) | 0.907 |
| **rs12708492** | -0.001 (-0.091 - 0.088) | 0.974 | 0.004 (-0.073 - 0.081) | 0.912 |
| **rs4776344** | -0.04 (-0.172 - 0.091) | 0.547 | -0.004 (-0.118 - 0.11) | 0.945 |
| **rs7162912** | -0.008 (-0.103 - 0.088) | 0.876 | 0.002 (-0.08 - 0.084) | 0.962 |
| **rs12102171** | -0.005 (-0.123 - 0.112) | 0.932 | 0.002 (-0.1 - 0.103) | 0.976 |
| **rs10518707** | 0.01 (-0.078 - 0.098) | 0.819 | -0.001 (-0.077 - 0.075) | 0.981 |

N: Number of individuals with genotyping data for each SNP, CI: Confidence Interval

Table S2- Univariate and multivariate linear regression for total osteophytes score and each SNP

|  | **Univariate Analysis** | | **Multivariate Analysis** | |
| --- | --- | --- | --- | --- |
| **SNP** | **Coefficient (95% CI)** | **P-value** | **Coefficient (95% CI)** | **P-value** |
| **rs3825977** | 0.143 (0.031 - 0.255) | 0.012 | 0.106 (0.01 - 0.201) | 0.03 |
| **rs2118612** | -0.119 (-0.235 - -0.003) | 0.044 | -0.089 (-0.188 - 0.01) | 0.078 |
| **rs6494629** | -0.094 (-0.181 - -0.007) | 0.034 | -0.062 (-0.137 - 0.012) | 0.100 |
| **rs12914140** | -0.103 (-0.28 - 0.074) | 0.253 | -0.101 (-0.252 - 0.049) | 0.187 |
| **rs16950687** | 0.048 (-0.049 - 0.144) | 0.333 | 0.044 (-0.038 - 0.126) | 0.296 |
| **rs3809572** | -0.046 (-0.18 - 0.088) | 0.499 | -0.058 (-0.172 - 0.056) | 0.320 |
| **rs11637581** | 0.023 (-0.071 - 0.116) | 0.635 | 0.034 (-0.046 - 0.113) | 0.405 |
| **rs2053295** | 0.083 (-0.05 - 0.215) | 0.222 | 0.047 (-0.066 - 0.161) | 0.411 |
| **rs2053294** | 0.076 (-0.058 - 0.209) | 0.268 | 0.045 (-0.069 - 0.16) | 0.435 |
| **rs7359174** | -0.1 (-0.24 - 0.039) | 0.159 | -0.047 (-0.166 - 0.072) | 0.436 |
| **rs11631839** | 0.042 (-0.046 - 0.129) | 0.350 | 0.029 (-0.045 - 0.104) | 0.442 |
| **rs745103** | -0.028 (-0.115 - 0.058) | 0.520 | -0.029 (-0.102 - 0.045) | 0.444 |
| **rs11637659** | -0.024 (-0.138 - 0.09) | 0.683 | -0.033 (-0.13 - 0.064) | 0.501 |
| **rs12900401** | -0.116 (-0.323 - 0.091) | 0.270 | -0.06 (-0.236 - 0.117) | 0.507 |
| **rs7183244** | -0.038 (-0.126 - 0.051) | 0.403 | -0.022 (-0.097 - 0.053) | 0.569 |
| **rs12708492** | 0.018 (-0.072 - 0.108) | 0.691 | 0.023 (-0.053 - 0.1) | 0.549 |
| **rs1470002** | 0.06 (-0.033 - 0.153) | 0.208 | 0.021 (-0.058 - 0.101) | 0.600 |
| **rs2278545** | -0.031 (-0.179 - 0.117) | 0.679 | -0.033 (-0.158 - 0.093) | 0.612 |
| **rs12901499** | -0.014 (-0.102 - 0.075) | 0.763 | -0.021 (-0.097 - 0.054) | 0.583 |
| **rs2118610** | 0.015 (-0.073 - 0.103) | 0.737 | 0.019 (-0.056 - 0.095) | 0.619 |
| **rs6494633** | 0.013 (-0.075 - 0.101) | 0.768 | 0.019 (-0.057 - 0.095) | 0.621 |
| **rs893473** | -0.045 (-0.162 - 0.071) | 0.446 | -0.027 (-0.127 - 0.072) | 0.589 |
| **rs7181878** | -0.04 (-0.127 - 0.048) | 0.376 | -0.017 (-0.092 - 0.058) | 0.664 |
| **rs718663** | -0.004 (-0.21 - 0.202) | 0.970 | -0.035 (-0.21 - 0.14) | 0.694 |
| **rs12913547** | 0.086 (-0.025 - 0.197) | 0.127 | 0.02 (-0.075 - 0.115) | 0.677 |
| **rs4601989** | 0.049 (-0.06 - 0.159) | 0.374 | 0.02 (-0.073 - 0.113) | 0.670 |
| **rs920293** | 0.031 (-0.115 - 0.176) | 0.678 | 0.026 (-0.099 - 0.15) | 0.686 |
| **rs10518707** | -0.001 (-0.09 - 0.089) | 0.986 | -0.014 (-0.09 - 0.062) | 0.724 |
| **rs1992215** | 0.002 (-0.093 - 0.097) | 0.970 | -0.014 (-0.095 - 0.067) | 0.737 |
| **rs12102171** | 0.014 (-0.105 - 0.133) | 0.822 | 0.017 (-0.084 - 0.118) | 0.741 |
| **rs11071939** | -0.056 (-0.221 - 0.108) | 0.500 | -0.024 (-0.164 - 0.116) | 0.736 |
| **rs17293443** | -0.002 (-0.107 - 0.103) | 0.969 | -0.013 (-0.102 - 0.076) | 0.767 |
| **rs744910** | -0.009 (-0.099 - 0.08) | 0.837 | -0.012 (-0.088 - 0.064) | 0.757 |
| **rs2289263** | 0.036 (-0.049 - 0.121) | 0.401 | 0.011 (-0.061 - 0.084) | 0.759 |
| **rs11071938** | 0.001 (-0.094 - 0.096) | 0.991 | -0.012 (-0.094 - 0.069) | 0.768 |
| **rs4147358** | -0.023 (-0.132 - 0.086) | 0.677 | -0.01 (-0.103 - 0.082) | 0.826 |
| **rs731874** | -0.034 (-0.125 - 0.057) | 0.465 | -0.007 (-0.085 - 0.071) | 0.861 |
| **rs7162912** | -0.002 (-0.098 - 0.095) | 0.972 | 0.007 (-0.075 - 0.09) | 0.862 |
| **rs4776344** | -0.042 (-0.175 - 0.091) | 0.537 | -0.009 (-0.123 - 0.104) | 0.874 |
| **rs12915039** | 0.008 (-0.101 - 0.116) | 0.891 | 0.006 (-0.087 - 0.099) | 0.896 |
| **rs11639295** | 0.029 (-0.069 - 0.126) | 0.563 | 0.001 (-0.082 - 0.084) | 0.988 |

N: Number of individuals with genotyping data for each SNP, CI: Confidence Interval

Table S3- Univariate and multivariate linear regression for total JSN score and each SNP

|  | **Univariate Analysis** | | **Multivaraite Analysis** | |
| --- | --- | --- | --- | --- |
| **SNP** | **Coefficient (95% CI)** | **P-value** | **Coefficient (95% CI)** | **P-value** |
| **rs3825977** | 0.222 (0.094 , 0.35) | 0.001 | 0.187 (0.066 , 0.309) | 0.003 |
| **rs11637581** | 0.058 (-0.05 , 0.166) | 0.292 | 0.068 (-0.033 , 0.17) | 0.186 |
| **rs12914140** | -0.124 (-0.327 , 0.08) | 0.233 | -0.126 (-0.317 , 0.066) | 0.199 |
| **rs745103** | -0.063 (-0.163 , 0.037) | 0.215 | -0.058 (-0.152 , 0.036) | 0.227 |
| **rs12900401** | -0.148 (-0.387 , 0.092) | 0.226 | -0.136 (-0.361 , 0.09) | 0.237 |
| **rs2118612** | -0.096 (-0.23 , 0.039) | 0.163 | -0.075 (-0.201 , 0.052) | 0.246 |
| **rs17293443** | -0.052 (-0.172 , 0.068) | 0.394 | -0.063 (-0.176 , 0.05) | 0.276 |
| **rs4601989** | 0.084 (-0.042 , 0.21) | 0.192 | 0.06 (-0.059 , 0.18) | 0.319 |
| **rs1470002** | 0.09 (-0.017 , 0.197) | 0.1 | 0.051 (-0.05 , 0.153) | 0.322 |
| **rs7359174** | -0.112 (-0.274 , 0.049) | 0.172 | -0.076 (-0.229 , 0.076) | 0.326 |
| **rs2118610** | 0.034 (-0.067 , 0.135) | 0.505 | 0.041 (-0.055 , 0.138) | 0.398 |
| **rs6494633** | 0.032 (-0.07 , 0.133) | 0.541 | 0.04 (-0.057 , 0.136) | 0.42 |
| **rs4147358** | -0.061 (-0.187 , 0.064) | 0.338 | -0.048 (-0.167 , 0.071) | 0.43 |
| **rs12102171** | -0.056 (-0.193 , 0.082) | 0.426 | -0.049 (-0.178 , 0.081) | 0.46 |
| **rs12708492** | 0.034 (-0.07 , 0.138) | 0.516 | 0.035 (-0.063 , 0.133) | 0.482 |
| **rs731874** | -0.059 (-0.165 , 0.046) | 0.269 | -0.036 (-0.136 , 0.065) | 0.487 |
| **rs7162912** | 0.022 (-0.09 , 0.134) | 0.698 | 0.037 (-0.069 , 0.142) | 0.497 |
| **rs6494629** | -0.047 (-0.148 , 0.053) | 0.357 | -0.029 (-0.125 , 0.066) | 0.551 |
| **rs718663** | -0.043 (-0.282 , 0.195) | 0.722 | -0.067 (-0.291 , 0.157) | 0.557 |
| **rs2053295** | 0.055 (-0.098 , 0.209) | 0.478 | 0.042 (-0.102 , 0.187) | 0.566 |
| **rs11071939** | -0.077 (-0.269 , 0.114) | 0.426 | -0.052 (-0.232 , 0.128) | 0.568 |
| **rs2278545** | 0.037 (-0.133 , 0.208) | 0.667 | 0.036 (-0.125 , 0.196) | 0.661 |
| **rs2289263** | 0.044 (-0.053 , 0.142) | 0.374 | 0.02 (-0.073 , 0.112) | 0.677 |
| **rs2053294** | 0.036 (-0.119 , 0.192) | 0.644 | 0.027 (-0.119 , 0.174) | 0.715 |
| **rs12915039** | 0.031 (-0.094 , 0.156) | 0.624 | 0.021 (-0.097 , 0.139) | 0.726 |
| **rs11637659** | -0.013 (-0.145 , 0.119) | 0.849 | -0.02 (-0.144 , 0.105) | 0.755 |
| **rs16950687** | 0.023 (-0.089 , 0.134) | 0.689 | 0.015 (-0.09 , 0.12) | 0.779 |
| **rs744910** | -0.008 (-0.111 , 0.095) | 0.879 | -0.013 (-0.11 , 0.085) | 0.798 |
| **rs12913547** | 0.067 (-0.061 , 0.195) | 0.307 | 0.015 (-0.106 , 0.136) | 0.809 |
| **rs4776344** | -0.013 (-0.167 , 0.142) | 0.873 | 0.016 (-0.13 , 0.162) | 0.83 |
| **rs11639295** | 0.034 (-0.079 , 0.147) | 0.558 | 0.011 (-0.096 , 0.117) | 0.843 |
| **rs1992215** | 0.009 (-0.101 , 0.118) | 0.875 | -0.01 (-0.114 , 0.093) | 0.846 |
| **rs11071938** | 0.007 (-0.103 , 0.116) | 0.905 | -0.01 (-0.114 , 0.094) | 0.85 |
| **rs7181878** | -0.018 (-0.119 , 0.083) | 0.728 | -0.003 (-0.099 , 0.093) | 0.948 |
| **rs893473** | -0.014 (-0.149 , 0.121) | 0.839 | -0.004 (-0.131 , 0.123) | 0.956 |
| **rs11631839** | 0.009 (-0.092 , 0.11) | 0.86 | -0.002 (-0.097 , 0.093) | 0.968 |
| **rs7183244** | -0.016 (-0.119 , 0.087) | 0.758 | 0.002 (-0.096 , 0.099) | 0.97 |
| **rs920293** | -0.004 (-0.172 , 0.164) | 0.965 | 0.003 (-0.156 , 0.162) | 0.971 |
| **rs12901499** | 0 (-0.103 , 0.103) | 0.998 | -0.001 (-0.098 , 0.096) | 0.977 |
| **rs3809572** | 0.004 (-0.151 , 0.16) | 0.958 | 0.001 (-0.145 , 0.148) | 0.985 |
| **rs10518707** | 0.006 (-0.098 , 0.109) | 0.911 | 0.001 (-0.097 , 0.098) | 0.988 |

N: Number of individuals with genotyping data for each SNP, CI: Confidence Interval

Table S4- Univariate and multivariate logistic regression for GOA and each SNP

|  | **Univariate Analysis** | | **Multivariate Analysis** | | **MAF** | |
| --- | --- | --- | --- | --- | --- | --- |
| **SNP** | **OR (95% CI)** | **P-value** | **OR (95% CI)** | **P-value** | **Controls** | **GOA** |
| **rs3825977** | 1.48 (1.08 - 2.03) | 0.016 | 1.47 (1.02 - 2.1) | 0.037 | 0.17 | 0.23 |
| **rs12914140** | 0.62 (0.36 - 1.05) | 0.076 | 0.53 (0.28 - 0.98) | 0.044 | 0.08 | 0.05 |
| **rs11637581** | 1.2 (0.93 - 1.57) | 0.166 | 1.36 (1.00 - 1.84) | 0.05 | 0.28 | 0.32 |
| **rs745103** | 0.82 (0.64 - 1.05) | 0.116 | 0.77 (0.58 - 1.02) | 0.068 | 0.47 | 0.42 |
| **rs2118612** | 0.71 (0.5 - 0.99) | 0.044 | 0.71 (0.48 - 1.05) | 0.087 | 0.2 | 0.15 |
| **rs7359174** | 0.67 (0.44 - 1.01) | 0.057 | 0.7 (0.44 - 1.14) | 0.153 | 0.13 | 0.09 |
| **rs3809572** | 0.86 (0.59 - 1.26) | 0.438 | 0.75 (0.48 - 1.18) | 0.212 | 0.13 | 0.11 |
| **rs2278545** | 0.79 (0.51 - 1.2) | 0.269 | 0.74 (0.45 - 1.2) | 0.224 | 0.11 | 0.09 |
| **rs893473** | 0.8 (0.57 - 1.12) | 0.197 | 0.79 (0.53 - 1.18) | 0.247 | 0.19 | 0.16 |
| **rs2118610** | 1.1 (0.86 - 1.41) | 0.451 | 1.18 (0.89 - 1.58) | 0.25 | 0.48 | 0.51 |
| **rs11071938** | 0.93 (0.71 - 1.21) | 0.579 | 0.83 (0.61 - 1.14) | 0.254 | 0.31 | 0.3 |
| **rs6494633** | 1.09 (0.85 - 1.4) | 0.496 | 1.18 (0.88 - 1.57) | 0.269 | 0.48 | 0.5 |
| **rs11071939** | 1.14 (0.72 - 1.82) | 0.568 | 1.34 (0.79 - 2.29) | 0.279 | 0.07 | 0.08 |
| **rs1992215** | 0.94 (0.72 - 1.23) | 0.659 | 0.84 (0.62 - 1.15) | 0.285 | 0.31 | 0.3 |
| **rs6494629** | 0.8 (0.62 - 1.02) | 0.075 | 0.86 (0.65 - 1.14) | 0.3 | 0.51 | 0.45 |
| **rs17293443** | 0.92 (0.69 - 1.24) | 0.600 | 0.87 (0.62 - 1.23) | 0.434 | 0.24 | 0.23 |
| **rs7162912** | 0.91 (0.7 - 1.2) | 0.521 | 0.9 (0.65 - 1.23) | 0.492 | 0.37 | 0.35 |
| **rs744910** | 0.93 (0.72 - 1.2) | 0.590 | 0.91 (0.68 - 1.21) | 0.502 | 0.49 | 0.47 |
| **rs1470002** | 1.03 (0.8 - 1.34) | 0.804 | 0.9 (0.66 - 1.22) | 0.506 | 0.37 | 0.38 |
| **rs731874** | 0.99 (0.77 - 1.29) | 0.96 | 1.1 (0.82 - 1.48) | 0.524 | 0.3 | 0.3 |
| **rs12900401** | 0.76 (0.42 - 1.37) | 0.358 | 0.8 (0.4 - 1.61) | 0.536 | 0.06 | 0.04 |
| **rs11637659** | 0.93 (0.67 - 1.29) | 0.665 | 0.89 (0.62 - 1.29) | 0.551 | 0.19 | 0.18 |
| **rs2289263** | 1.02 (0.8 - 1.29) | 0.893 | 0.92 (0.7 - 1.22) | 0.573 | 0.47 | 0.48 |
| **rs12901499** | 0.98 (0.76 - 1.25) | 0.846 | 0.92 (0.69 - 1.23) | 0.576 | 0.48 | 0.47 |
| **rs11631839** | 1.11 (0.87 - 1.42) | 0.414 | 1.07 (0.81 - 1.42) | 0.629 | 0.46 | 0.49 |
| **rs10518707** | 1 (0.78 - 1.28) | 0.993 | 0.93 (0.7 - 1.25) | 0.637 | 0.48 | 0.48 |
| **rs718663** | 0.96 (0.53 - 1.74) | 0.902 | 0.85 (0.43 - 1.69) | 0.645 | 0.05 | 0.05 |
| **rs920293** | 1.11 (0.74 - 1.67) | 0.625 | 1.11 (0.69 - 1.78) | 0.664 | 0.09 | 0.1 |
| **rs4776344** | 0.95 (0.65 - 1.39) | 0.806 | 1.07 (0.7 - 1.66) | 0.749 | 0.13 | 0.13 |
| **rs7183244** | 0.91 (0.71 - 1.17) | 0.463 | 0.95 (0.72 - 1.27) | 0.754 | 0.41 | 0.39 |
| **rs2053294** | 1.14 (0.78 - 1.66) | 0.503 | 1.06 (0.69 - 1.64) | 0.794 | 0.12 | 0.13 |
| **rs12102171** | 1.02 (0.73 - 1.43) | 0.905 | 1.04 (0.71 - 1.53) | 0.845 | 0.16 | 0.16 |
| **rs11639295** | 1.11 (0.85 - 1.46) | 0.447 | 1.03 (0.75 - 1.41) | 0.851 | 0.29 | 0.31 |
| **rs7181878** | 0.94 (0.73 - 1.2) | 0.612 | 1.02 (0.77 - 1.36) | 0.867 | 0.5 | 0.48 |
| **rs4601989** | 1.07 (0.79 - 1.46) | 0.653 | 0.97 (0.69 - 1.38) | 0.881 | 0.2 | 0.22 |
| **rs4147358** | 0.95 (0.69 - 1.29) | 0.733 | 0.98 (0.68 - 1.4) | 0.895 | 0.21 | 0.2 |
| **rs12915039** | 1.01 (0.75 - 1.38) | 0.925 | 0.98 (0.69 - 1.4) | 0.911 | 0.22 | 0.22 |
| **rs16950687** | 1.03 (0.79 - 1.35) | 0.827 | 1.02 (0.75 - 1.39) | 0.911 | 0.28 | 0.28 |
| **rs12708492** | 0.98 (0.76 - 1.27) | 0.905 | 0.99 (0.74 - 1.33) | 0.958 | 0.48 | 0.48 |
| **rs12913547** | 1.19 (0.87 - 1.63) | 0.266 | 0.99 (0.69 - 1.42) | 0.965 | 0.19 | 0.22 |
| **rs2053295** | 1.11 (0.76 - 1.61) | 0.582 | 1.01 (0.66 - 1.55) | 0.965 | 0.13 | 0.14 |

OR: Odds Ratio, CI: Confidence Interval, MAF: Minor allele frequency
